# Supplementary material for: High type I collagen density fails to increase breast cancer stem cell phenotype
Source: PeerJ. 2020 May 12;8:e9153. doi: 10.7717/peerj.9153 (PMC7227653; doi:10.7717/peerj.9153)
Supplement: Supplemental Information 1 — FCS-A vs. SSC-A (A) and FCS-A vs. FCS-W (B) dot plots were used to exclude cell debris and cell clumps, respectively. FITC vs. PE dot plots of gated viable singlets were then generated (C) and percentage of population of interest (FITC+/PE-) was obtained (D). [file peerj-08-9153-s001.pdf]

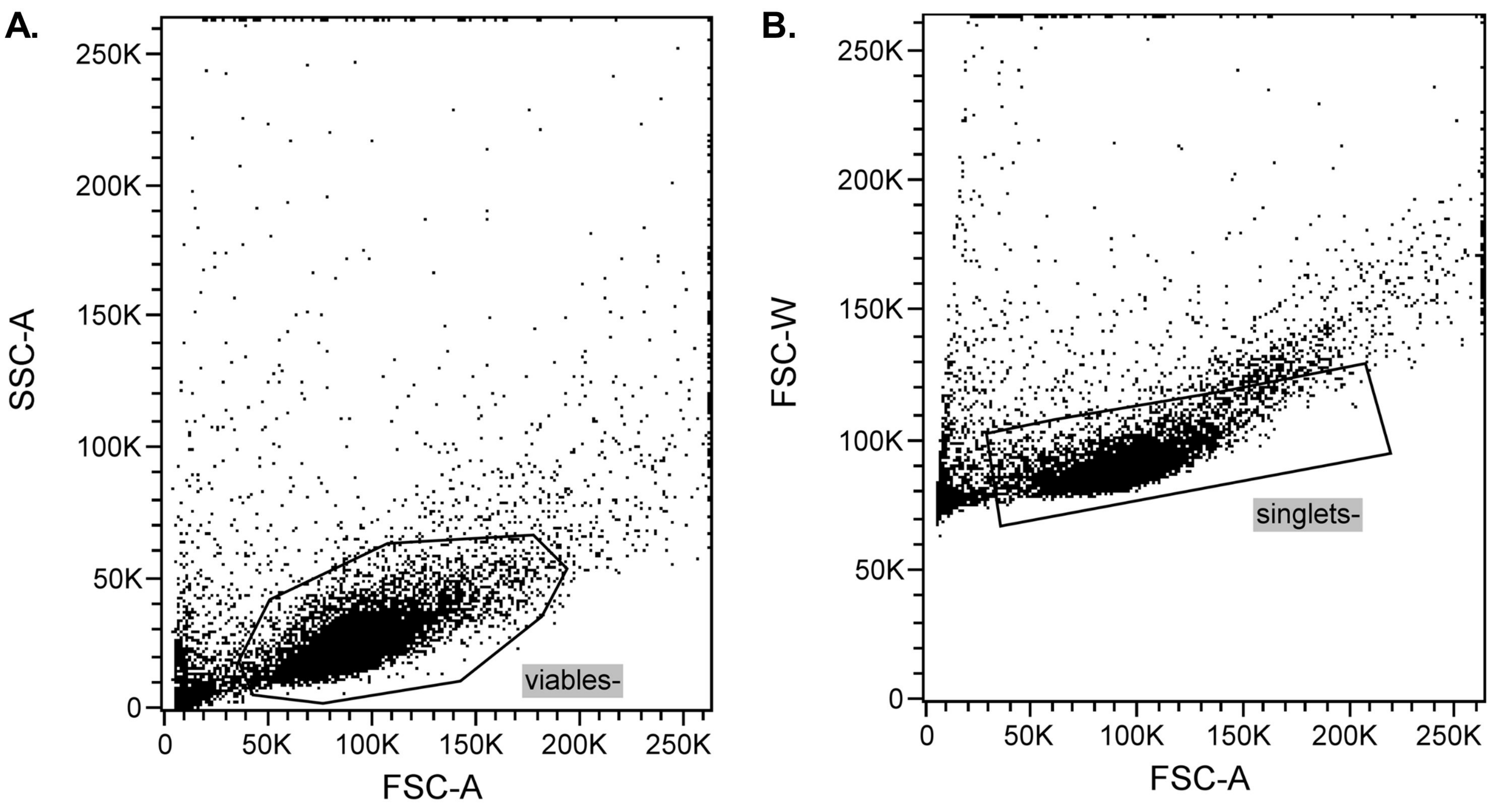

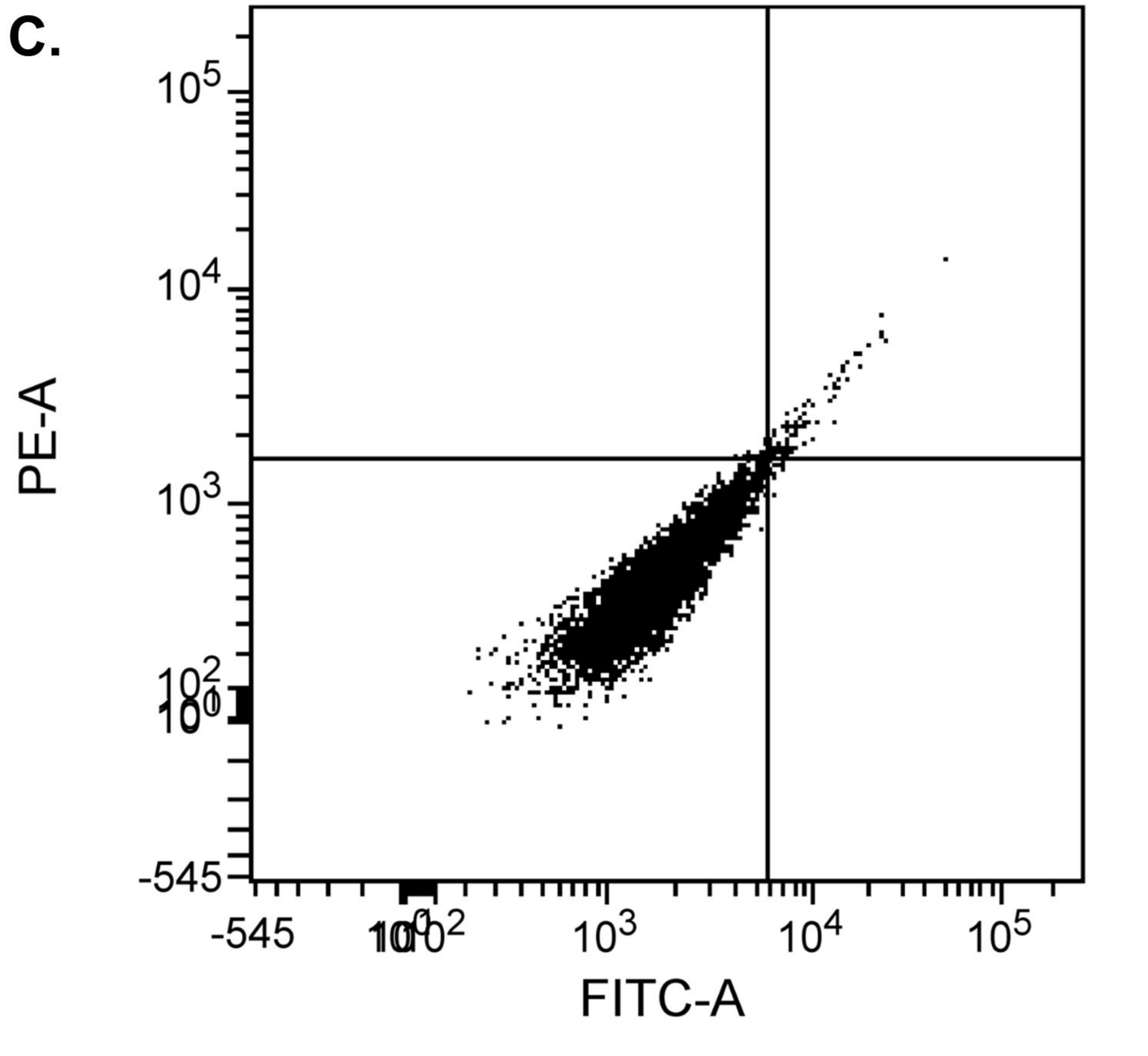

D.

Total events: 10000
Gate: viables and singles

Label	Events	Percent gated
all	7270	100.00
FITC-A- PE-A-	7141	98.23
FITC-A+ PE-A-	20	0.28
FITC-A- PE-A+	20	0.28
FITC-A+ PE-A+	89	1.22
