# Supplementary figures and images for: High type I collagen density fails to increase breast cancer stem cell phenotype
Source: PeerJ. 2020 May 12;8:e9153. doi: 10.7717/peerj.9153 (PMC7227653; doi:10.7717/peerj.9153)

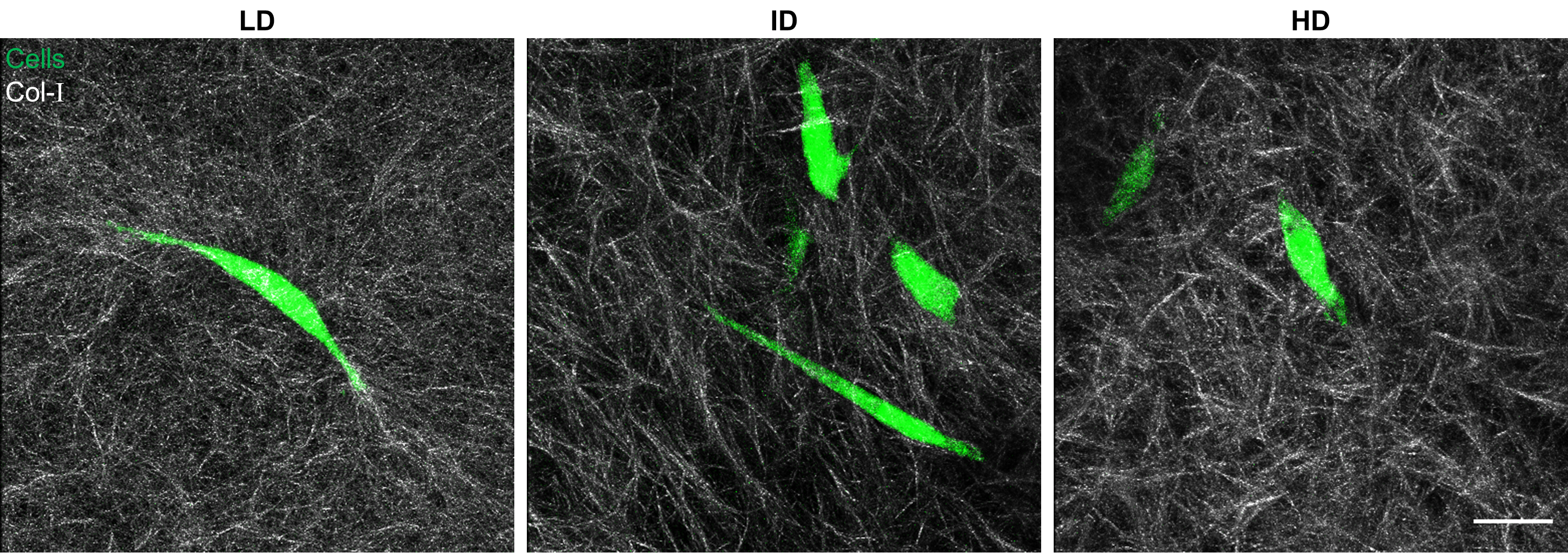

Supplement: Supplemental Information 2 — Col-I fibers were imaged by confocal reflectance (grey) while cells were stained with CellTracker Green (green) right before image acquisition. Representative cell morphology for each of the Col-I densities is presented. Scale bar: 25 μm. [file peerj-08-9153-s002.png]
