# Supplementary material for: High type I collagen density fails to increase breast cancer stem cell phenotype
Source: PeerJ. 2020 May 12;8:e9153. doi: 10.7717/peerj.9153 (PMC7227653; doi:10.7717/peerj.9153)
Supplement: Supplemental Information 3 [file peerj-08-9153-s003.docx]

Supplemental Table 1:

List of gene array fold change regulation relative to 2D group

| Plate position | Refseq | Gene  (symbol) | Col-I density | | | Comments |
| --- | --- | --- | --- | --- | --- | --- |
|  |  |  | LD | ID | HD |  |
|  |  |  | Fold change (relative to 2D) | | |  |
| A01 | NM_178559 | ABCB5 | 5.6834 | 5.5189 | 5.4745 | C |
| A02 | NM_004827 | ABCG2 | 0.5071 | 0.1235 | 0.4485 | B |
| A03 | NM_001627 | ALCAM | 0.7727 | 0.7998 | 0.7377 | OKAY |
| A04 | NM_000689 | ALDH1A1 | 5.0944 | 0.5476 | 2.3082 | B |
| A05 | NM_000051 | ATM | 3.2318 | 2.6416 | 2.0199 | OKAY |
| A06 | NM_000332 | ATXN1 | 3.4481 | 3.9224 | 2.4147 | OKAY |
| A07 | NM_001699 | AXL | 1.1149 | 1.4571 | 1.2845 | OKAY |
| A08 | NM_005180 | BMI1 | 1.0967 | 0.9989 | 0.9749 | OKAY |
| A09 | NM_001719 | BMP7 | 5.6834 | 5.5189 | 5.4745 | C |
| A10 | NM_013230 | CD24 | 49.3261 | 25.3935 | 29.7586 | B |
| A11 | NM_001773 | CD34 | 5.6834 | 5.5189 | 5.4745 | C |
| A12 | NM_001775 | CD38 | 2.8076 | 0.2616 | 0.2595 | B |
| B01 | NM_000610 | CD44 | 3.2322 | 3.7338 | 3.9291 | OKAY |
| B02 | NM_001274 | CHEK1 | 0.7498 | 1.1234 | 0.8167 | OKAY |
| B03 | NM_004392 | DACH1 | 0.0724 | 0.0577 | 0.0536 | A |
| B04 | NM_001954 | DDR1 | 0.5331 | 0.5873 | 0.2919 | A |
| B05 | NM_012242 | DKK1 | 0.1891 | 0.2432 | 0.2732 | OKAY |
| B06 | NM_005618 | DLL1 | 0.7802 | 0.5971 | 0.1379 | B |
| B07 | NM_019074 | DLL4 | 9.8022 | 9.7474 | 5.3783 | B |
| B08 | NM_001379 | DNMT1 | 0.2637 | 0.6191 | 0.3002 | OKAY |
| B09 | NM_001963 | EGF | 0.5334 | 0.4784 | 1.0389 | B |
| B10 | NM_000118 | ENG | 2.2413 | 6.0592 | 1.7641 | B |
| B11 | NM_002354 | EPCAM | 0.8024 | 1.079 | 1.0096 | OKAY |
| B12 | NM_004448 | ERBB2 | 0.348 | 0.8473 | 0.3354 | A |
| C01 | NM_000126 | ETFA | 0.9551 | 1.1515 | 1.2077 | OKAY |
| C02 | NM_000141 | FGFR2 | 5.6834 | 5.5189 | 5.4745 | C |
| C03 | NM_004475 | FLOT2 | 0.5744 | 0.7381 | 0.5654 | OKAY |
| C04 | NM_021784 | FOXA2 | 0.5179 | 0.6617 | 0.4959 | OKAY |
| C05 | NM_032682 | FOXP1 | 1.1316 | 0.6102 | 0.6542 | OKAY |
| C06 | NM_003507 | FZD7 | 0.7072 | 0.8116 | 0.3986 | OKAY |
| C07 | NM_002051 | GATA3 | 0.1261 | 0.2551 | 0.3223 | B |
| C08 | NM_002093 | GSK3B | 1.1913 | 0.967 | 1.0171 | OKAY |
| C09 | NM_004964 | HDAC1 | 0.4373 | 0.4105 | 0.4379 | OKAY |
| C10 | NM_002165 | ID1 | 4.2998 | 3.0475 | 3.9642 | OKAY |
| C11 | NM_001556 | IKBKB | 1.3511 | 1.3422 | 1.2539 | OKAY |
| C12 | NM_000584 | CXCL8 | 1.9588 | 2.012 | 1.962 | OKAY |
| D01 | NM_002203 | ITGA2 | 1.3403 | 1.9542 | 1.4105 | OKAY |
| D02 | NM_000885 | ITGA4 | 5.6834 | 5.5189 | 5.4745 | C |
| D03 | NM_000210 | ITGA6 | 1.8589 | 2.3049 | 2.3618 | OKAY |
| \| \| Plate position \| Refseq \| Gene  (symbol) \| Coll I density \| \| \| Comments \| \| --- \| --- \| --- \| --- \| --- \| --- \| --- \| \| LD \| ID \| HD \| \| Fold change (relative to 2D) \| \| \| \| \| --- \| --- \| --- \| --- \| --- \| --- \| --- \| --- \| --- \| --- \| --- \| --- \| --- \| --- \| | | | | | | |
| D04 | NM_002211 | ITGB1 | 0.653 | 0.8002 | 0.6863 | OKAY |
| D05 | NM_000214 | JAG1 | 1.6908 | 1.1482 | 1.3051 | OKAY |
| D06 | NM_004972 | JAK2 | 0.9955 | 0.5054 | 0.5731 | A |
| D07 | NM_000222 | KIT | 5.6834 | 5.5189 | 5.4745 | C |
| D08 | NM_003994 | KITLG | 0.021 | 0.1125 | 0.0202 | A |
| D09 | NM_173484 | KLF17 | 9.5777 | 1.3509 | 4.6123 | B |
| D10 | NM_004235 | KLF4 | 1.1534 | 0.9604 | 0.9439 | OKAY |
| D11 | NM_004690 | LATS1 | 1.2668 | 0.8959 | 0.9358 | OKAY |
| D12 | NM_024674 | LIN28A | 34.0645 | 15.7398 | 22.4146 | B |
| E01 | NM_00100431 | LIN28B | 5.6834 | 5.5189 | 5.4745 | C |
| E02 | NM_014757 | MAML1 | 0.3962 | 0.3819 | 0.3235 | A |
| E03 | NM_006343 | MERTK | 0.1681 | 0.2282 | 0.1591 | OKAY |
| E04 | NM_021950 | MS4A1 | 5.6834 | 5.5189 | 5.4745 | C |
| E05 | NM_00101801 | MUC1 | 2.3148 | 1.841 | 1.1913 | OKAY |
| E06 | NM_002467 | MYC | 0.4881 | 0.5097 | 0.5105 | OKAY |
| E07 | NM_005378 | MYCN | 5.6834 | 5.5189 | 5.4745 | C |
| E08 | NM_024865 | NANOG | 105.6878 | 24.0278 | 40.5926 | B |
| E09 | NM_003998 | NFKB1 | 0.3304 | 0.3171 | 0.2995 | A |
| E10 | NM_000625 | NOS2 | 2.8291 | 1.0932 | 2.6347 | B |
| E11 | NM_017617 | NOTCH1 | 1.7953 | 2.3226 | 1.4867 | A |
| E12 | NM_024408 | NOTCH2 | 2.2178 | 2.4478 | 1.5079 | OKAY |
| F01 | NM_000442 | PECAM1 | 1.7502 | 0.8969 | 1.3685 | B |
| F02 | NM_000930 | PLAT | 3.3842 | 2.8615 | 3.2684 | OKAY |
| F03 | NM_002659 | PLAUR | 9.4456 | 9.9732 | 9.7354 | OKAY |
| F04 | NM_002701 | POU5F1 | 23.3705 | 66.4514 | 19.8901 | B |
| F05 | NM_006017 | PROM1 | 1.2866 | 1.2494 | 1.2393 | B |
| F06 | NM_000264 | PTCH1 | 0.0645 | 0.0632 | 0.0967 | B |
| F07 | NM_002838 | PTPRC | 5.6834 | 5.5189 | 5.4745 | C |
| F08 | NM_021818 | SAV1 | 0.8414 | 0.6336 | 0.6544 | OKAY |
| F09 | NM_012238 | SIRT1 | 0.6858 | 0.5566 | 0.6518 | OKAY |
| F10 | NM_005631 | SMO | 5.6834 | 5.5189 | 5.4745 | C |
| F11 | NM_005985 | SNAI1 | 5.2018 | 6.112 | 4.7397 | B |
| F12 | NM_003106 | SOX2 | 0.22 | 0.8498 | 0.0529 | B |
| G01 | NM_003150 | STAT3 | 0.8223 | 0.768 | 0.6445 | OKAY |
| G02 | NM_000116 | TAZ | 0.6157 | 0.6691 | 0.3664 | OKAY |
| G03 | NM_004612 | TGFBR1 | 0.6049 | 0.5007 | 0.5613 | OKAY |
| G04 | NM_006288 | THY1 | 5.6834 | 5.5189 | 5.4745 | C |
| G05 | NM_000474 | TWIST1 | 5.6834 | 5.5189 | 5.4745 | C |
| G06 | NM_057179 | TWIST2 | 24.0529 | 5.5189 | 5.4745 | C |
| G07 | NM_003390 | WEE1 | 0.6418 | 0.7584 | 0.5046 | OKAY |
| G08 | NM_005430 | WNT1 | 2.6525 | 2.5757 | 2.555 | B |
| G09 | NM_015238 | WWC1 | 0.186 | 0.3392 | 0.3503 | A |
| \| \| Plate position \| Refseq \| Gene  (symbol) \| Coll I density \| \| \| Comments \| \| --- \| --- \| --- \| --- \| --- \| --- \| --- \| \| LD \| ID \| HD \| \| Fold change (relative to 2D) \| \| \| \| \| --- \| --- \| --- \| --- \| --- \| --- \| --- \| --- \| --- \| --- \| --- \| --- \| --- \| --- \| | | | | | | |
| G10 | NM_006106 | YAP1 | 0.458 | 0.5483 | 0.429 | OKAY |
| G11 | NM_030751 | ZEB1 | 0.8204 | 0.7788 | 0.6229 | OKAY |
| G12 | NM_014795 | ZEB2 | 3.7462 | 4.5587 | 3.6373 | OKAY |
| H01 | NM_001101 | ACTB | 0.7402 | 0.6844 | 0.7424 | OKAY |
| H02 | NM_004048 | B2M | 0.807 | 0.7678 | 0.7983 | OKAY |
| H03 | NM_002046 | GAPDH | 1.608 | 2.0345 | 1.3893 | OKAY |
| H04 | NM_000194 | HPRT1 | 0.9596 | 1.0102 | 1.091 | OKAY |
| H05 | NM_001002 | RPLP0 | 1.0851 | 0.926 | 1.1132 | OKAY |

Comments:

**A**: This gene’s average threshold cycle is relatively high (> 30) in either the control or the test sample, and is reasonably low in the other sample (< 30). These data mean that the gene’s expression is relatively low in one sample and reasonably detected in the other sample suggesting that the actual fold-change value is at least as large as the calculated and reported fold-change result. This fold-change result may also have greater variations if p value > 0.05; therefore, it is important to have a sufficient number of biological replicates to validate the result for this gene.

**B**: This gene’s average threshold cycle is relatively high (> 30), meaning that its relative expression level is low, in both control and test samples, and the p-value for the fold-change is either unavailable or relatively high (p > 0.05). This fold-change result may also have greater variations; therefore, it is important to have a sufficient number of biological replicates to validate the result for this gene.

**C**: This gene’s average threshold cycle is either not determined or greater than the defined cut-off value (default 35), in both samples meaning that its expression was undetected, making this fold-change result erroneous and un-interpretable.
