# Supplementary material for: High type I collagen density fails to increase breast cancer stem cell phenotype
Source: PeerJ. 2020 May 12;8:e9153. doi: 10.7717/peerj.9153 (PMC7227653; doi:10.7717/peerj.9153)
Supplement: Supplemental Information 4 [file peerj-08-9153-s004.docx]

Supplemental Table 2:

| List of primers used in this study | | | |
| --- | --- | --- | --- |
| Target gene | Forward sequence (5’-3’) | Reverse sequence (5’-3’) | Product  length (bp) |
| *NANOG* | TTTGTGGGCCTGAAGAAAACT | AGGGCTGTCCTGAATAAGCAG | 116 |
| *POU5F1/OCT4* | GTGTTCAGCCAAAAGACCATCT | GGCCTGCATGAGGGTTTCT | 156 |
| *SNAI1* | TAGCGAGTGGTTCTTCTGCG | TTAGGCTTCCGATTGGGGTC | 114 |
| *GAPDH* | ACCCACTCCTCCACCTTTGA | CTGTTGCTGTAGCCAAATTCGT | 101 |
