# Supplementary material for: High type I collagen density fails to increase breast cancer stem cell phenotype
Source: PeerJ. 2020 May 12;8:e9153. doi: 10.7717/peerj.9153 (PMC7227653; doi:10.7717/peerj.9153)
Supplement: Supplemental Information 5 [file peerj-08-9153-s005.docx]

| Antibodies | Host | Source | Catalog number | Dilution | | |
| --- | --- | --- | --- | --- | --- | --- |
|  |  |  |  | IB | IF | |
|  |  |  |  |  | 2D, MS | Col-I |
| β-Actin | Mouse | Santa Cruz | sc-47778 | 1:2000 |  |  |
| CD44 | Mouse | Cell Signaling | #3570 | 1:1000 | 1:200 | 1:100 |
| E-cadherin | Mouse | Cell Signalling | #14472 | 1:1000 | 1:200 | 1:100 |
|  |  | Santa Cruz | sc-8426 | 1:500 | 1:100 | 1:50 |
| Integrin-α6 | Rabbit | Cell Signaling | #3750 | 1:1000 |  |  |
| Ki-67 | Rabbit | Cell Signaling | #12202 |  |  | 1:200 |
| Mouse IgG (H+L),  Alexa Fluor 568 | Goat | ThermoFischer  Scientific | A-11004 |  | 1:500 | 1:250 |
| Mouse IgG (HL)-HRP | Goat | Bio-Rad | #172-1011 | 1:3000 |  |  |
| NANOG | Rabbit | Cell Signaling | #3580 | 1:1000 | 1:100 | 1:50 |
| PCNA | Mouse | Santa Cruz | sc-56 |  |  | 1:200 |
| PP2A | Mouse | Santa Cruz | sc-374380 | 1:500 |  |  |
| Rabbit IgG (H+L),  Alexa Fluor 568 | Goat | ThermoFischer  Scientific | A-11011 |  | 1:500 | 1:250 |
| Rabbit IgG (HL)-HRP | Goat | Bio-Rad | #172-1019 | 1:3000 |  |  |
| ERα | Mouse | Santa Cruz | sc-8002 | 1:1000 | 1:100 | 1:50 |
| SAV1 | Mouse | Santa Cruz | sc-374366 | 1:500 |  |  |
| Sox2 | Rabbit | Cell Signalling | #3579 | 1:500 | 1:100 | 1:50 |
| WBP2 | Mouse | Santa Cruz | sc-514247 | 1:500 |  |  |
| YAP | Mouse | Santa Cruz | sc-271134 | 1:500 | 1:100 | 1:50 |
|  |  | Santa Cruz | sc-376830 | 1:500 | 1:100 | 1:50 |
|  |  | Cell Signaling | #12395 | 1:1000 | 1:100 | 1:50 |

Supplemental Table 3:

List of antibodies used for IF and IB in the study

IB = immunoblotting, IF = immunofluorescence, 2D = bidimensional surface, MS = mammospheres, Col-I = Collagen I
